# Karl S. Matlin, *Crossing the Boundaries of Life: Günter Blobel and the Origins of Molecular Cell Biology*, Chicago: University of Chicago Press, 2022, ISBN: 9780226819235, 368 pp

**DOI:** 10.1007/s10739-023-09730-y

**Published:** 2023-07-27

**Authors:** Daniel Liu

**Affiliations:** Ludwig-Maximilians-Universität München, Munich, Germany

The last few years have seen a mini-boom of books on the history of modern molecular cell biology. Chief among these have been Andrew Reynolds’ excellent *The Third Lens* (2018; reviewed here by Karl Matlin in vol. 54, no. 3) and the volume *Visions of Cell Biology*, edited by Karl Matlin, Jane Maienschein, and Manfred Laubichler (2018); and, slightly farther afield, Mathias Grote’s *Membranes to Molecular Machines* (2019), and Sherrie Lyons’ *From Cells to Organisms* (2020; reviewed here by Garland Allen in vol. 55, no. 1). Previously, William Bechtel’s *Discovering Cell Mechanisms* (2006; reviewed here by Lindley Darden in vol. 40, no. 1) stood almost alone in providing a strong framework for exploring the history of post-1950 cell biology, joined in 2012 by Carol Moberg’s unjustly neglected *Entering an Unseen World*. At first glance, Karl Matlin’s latest book is a history of the signal hypothesis and its central author, Nobel laureate Günter Blobel (1936–2018). But *Crossing the Boundaries of Life* achieves much more than that: it is the biggest and most comprehensive history of cell biology to date, focused on the crucial period 1940–1980 and with coverage extending from 1837 out to 1990. Based solely on its originality, wealth of detail, and subject matter, *Crossing the Boundaries of Life* deserves to be on the must-read list of every historian of the twentieth-century life sciences. That it is also a dense and demanding book might explain why this has not been attempted before, and might be seen as a cautionary tale—or a warning shot—for historians, philosophers, and critics of post-1950 molecular biology.

The heart of *Crossing the Boundaries of Life* is Matlin’s detailed excavation of Blobel’s signal hypothesis (chaps. 5–9), which Blobel formulated in papers published in 1971, 1975, and 1980. Briefly, Blobel proposed that proteins destined for either secretion or insertion into the cell membrane are encoded with a short “signal sequence,” a chain of amino acids at a nascent protein’s N-terminus that causes the translating ribosome to bind to the endoplasmic reticulum and insert the growing polypeptide into the interior of the ER. By 1980 Blobel expanded this theory to propose that signal sequences encode “addresses” for every conceivable location in the cell—and, as it turned out, for every dynamic pathway in the cell as well. The exact mechanisms and molecular components for the signal hypothesis were worked out, and at times heatedly contested, by dozens of scientists working across laboratories in the United States, Britain, and the two Germanies. While Blobel is Matlin’s central ideas man, *Crossing the Boundaries of Life* is foremost a history of a scientific theory, along with the many investigative pathways that it joined together and the methods used to explicate it.

But *Crossing the Boundaries of Life* has larger ambitions. This Matlin’s biggest and (so far) best platform for arguing that modern, molecularized cell biology relies on what Matlin calls an “epistemic strategy” of linking morphological knowledge to either a) in vitro experimentation on cell fractions, b) in vitro experimentation on single proteins/mechanisms, or c) bioinformatic analyses of genomes, proteomes, etc. For all of the generativity of these three novel analytical methods, Matlin argues that their results only gain “biological meaning” when they are placed in a cellular “context” (p. 248). Put another way, Matlin argues that it has never been properly “biological” to only search for a biochemical pathway or mechanism, or, worse, to use only “grind and find” methods in biochemistry. For Matlin, the origin of cell biology and its key epistemic strategy—what makes cell biology fundamentally cellular and biological—dates to the 1930–50s, when Albert Claude (1899–1983), George Palade (1912–2008), and Keith Porter (1912–1997) took electron microscopy and cell culture and combined them with cell fractionation and quantitative biochemical analysis, to show that ER ribosomes synthesize proteins and mitochondria are the sites of oxidative phosphorylation (chaps. 3–4). The structure or *topology* of the cell was necessary foreknowledge to unpack how these mechanisms worked across membrane-bound compartments (chap. 9). Without a “heuristics of cellular form,” Matlin argues that we would be left only with mere “biochemical events” having neither order nor location. Given this, Blobel’s signal hypothesis represents the molecularization of cell biology’s core epistemic strategy, the translation of the cell’s spatial organization into genetic and amino acid code. Nor does this absolutely require an electron microscopist on staff. In chapter 10 Matlin shows how Blobel and others developed in vitro experimental systems that preserved enough cellular topology to do morphologically-informed cell biology—topogenesis in the test tube. Building on scholarship by Bill Bechtel and Nicolas Rasmussen, Matlin argues that in both theory and method, “cellular function cannot be considered apart from the cell’s spatial organization. Cell structure and function are not independent entities” (p. 237).

This is a highly opinionated book, and despite his detached writing style, Matlin is not merely a distant observer. Now emeritus professor at both the Department of Surgery and the Committee on Conceptual and Historical Studies of Science at University of Chicago, Matlin discloses in advance that he did his graduate studies in cell biology at the Rockefeller University in the 1970s, where “my mentor’s laboratory was located across the corridor from Günter Blobel’s laboratory” (p. *ix*). We get close glimpses of scientists’ human character (sometimes unflattering), a proximity owing to Matlin’s own physical and personal history: “Even though I was never a true member of [Blobel’s scientific] family, I was certainly an adopted child” (p. *x*). Regardless, the wealth of information and new stories should dispel most doubts about Matlin’s objectivity. Matlin started collecting interviews in 1998, including three with Blobel himself, and clear outlines of *Crossing the Boundaries of Life* appeared in *Nature Reviews Molecular Cell Biology* in 2011.^1^ Matlin’s experience as a cell biologist who worked on topogenesis helps him guide readers through the minutiae of experiments being done in rival laboratories. Future students of the history of cell biology will doubtless find this book a valuable fund of facts and anecdotes; the index is excellent. At times the red thread of the signal hypothesis has to go in loops and knots, as Matlin does his best to give a comprehensive narrative of a lot of scientists trying to solve different but overlapping problems, using a wide range of tools at their disposal.

Although readers who do not have a background in cell biology or biochemistry may feel themselves stymied by the sheer quantity of detail, Matlin’s strong and clear arguments will reward the tenacious. Besides, the detail is the point: in 1999 Blobel was the sole recipient of the Nobel Prize for the signal hypothesis, and Matlin’s extremely full picture of the experimental work of scientists illustrates how a singular achievement is still a communal endeavor. This is especially clear in chapters 7–8, where we get a view of Blobel’s ongoing competition with his former postdoc and coauthor Bernhard Dobberstein, as well as with Tom Rapoport, who leveraged sympathy for his circumstances in East Germany to learn about the Blobel lab’s experimental systems. We learn about sharp-elbowed postdocs like Flora Katz and James Rothman who eventually learn how to work together, as well as Blobel’s more acrimonious turf battles with Nam-Hai Chua, Gottfried Schatz, and Maria Luisa Maccecchini—rivalries that nevertheless generated important results. At times one strains to keep track of all of the people and their projects. The book admittedly also has literary flaws that make things more difficult: an editor should have caught problems with verb tense, the chronological timing of some events is hard to pin down, and explanations of some procedural terms (detergents, enrichment, high salt extraction) are only given pages after they are first introduced. And getting the most out of the book might require some homework. In addition to having some knowledge of the history of molecular genetics, I recommend reviewing Hans-Jörg Rheinberger’s 1997 *Toward a History of Epistemic Things* (and not just every other chapter, as some are wont to do), Nicolas Rasmussen’s *Picture Control* (1997), and skimming chapter 12 of Bruce Albert’s standard textbook *Molecular Biology of the Cell* (2015).

*Crossing the Boundaries of Life* is a challenge, and not just to read. For cell biologists reading this book, Matlin challenges his colleagues to accept that biochemical dynamics alone are insufficient, and that form and morphology are vital for understanding cellular mechanisms (chaps. 10–11). For historians of the modern life sciences, and in particular to habitual critics of mechanistic reductionism, Matlin’s challenge to his current colleagues—readers of this journal—is to appreciate and digest the richness of mechanistic laboratory biology. Indeed, at times I wondered if the history of the signal hypothesis is so complex that it needs a view from inside to get all the details right. But most of all, *Crossing the Boundaries of Life* makes the clearest argument thus far for what molecular cell biology is, and provides an exemplary case study of how cell biologists have worked.

## Footnotes

1 Matlin, Karl S. 2011. Spatial Expression of the Genome: The Signal Hypothesis at Forty. *Nature Reviews Molecular Cell Biology* 12: 333–40, 10.1038/nrm3105.

